# Influence of co-morbidity on body composition changes after weight loss intervention among overweight housewives: a follow-up study of the MyBFF@home

**DOI:** 10.1186/s12905-018-0600-6

**Published:** 2018-07-19

**Authors:** Nur Shahida Abdul Aziz, Suzana Shahar, Rashidah Ambak, Noor Safiza Mohamad Nor, Ahmad Taufik Jamil, Tahir Aris

**Affiliations:** 1 Institute for Public Health, National Institute of Health, Ministry of Health Malaysia, 50590 Kuala Lumpur, Malaysia; 2 Department of Nutrition and Dietetics, Faculty of Allied Health Sciences, Universiti Kebangsaan Malaysia, Kuala Lumpur, Malaysia; 3 Population Health and Preventive Medicine, Faculty of Medicine, University Technology MARA (UiTM), Sungai Buloh Campus, Sungai Buloh, Selangor Darul Ehsan Malaysia

**Keywords:** Co-morbidities, Body composition, Weight loss intervention, Women, Housewives

## Abstract

**Background:**

Obesity is a risk factor for co-morbidities such as diabetes, hypertension, osteoarthritis and cardiovascular diseases. However, it is unclear if the presence of co-morbidities has any effect on the magnitude of body composition changes following weight reduction programmes. Thus, this study aimed to determine changes in body composition among obese housewives with and without co-morbidities after they participated in a weight loss intervention.

**Methods:**

This is a follow-up study among 84 obese housewives without co-morbidities aged 18 to 59 years old who previously participated as a control group (delayed intervention, G1) in the My Body is Fit and Fabulous at Home (MyBFF@home) Phase II. Baseline data were obtained from 12 month data collection for this group. A new group of 42 obese housewives with co-morbidities (G2) were also recruited. Both groups received a 6 month intervention (July–December 2015) consisting of dietary counselling, physical activity (PA) and self-monitoring tools (PA diary, food diary and pedometer). Study parameters included weight, height, waist circumference, blood pressure and body compositions. Body compositions were measured using a bioelectrical impedance analysis device, Inbody 720. Descriptive and repeated measures ANOVA analyses were performed using SPSS 21.

**Results:**

There were reductions in mean body fat, fat mass and visceral fat area, particularly among obese women without co-morbidities. There were also decreases fat and skeletal muscle from baseline to month six with mean difference − 0.12 (95% CI: -0.38, 0.14) and visceral fat area from month three to month six with mean difference − 9.22 (− 17.87, − 0.56) for G1. G2 showed a decreasing pattern of skeletal muscle from baseline to month six with mean difference − 0.01(95% CI: -0.38, 0.37). There was a significant difference for group effect of visceral fat area (*p* < 0.05) with mean difference of − 11.49(95% CI: -20.07, 2.91). It showed that the intervention programs was effective to reduce visceral fat area compared to other part of body composition.

**Conclusion:**

Obese participants without co-morbidities showed more desirable changes in body composition. Visceral fat area was reduced regardless of morbidity status. Weight management efforts are therefore not as straightforward in those with co-morbidities compared to those without, and require thorough and tailor-made strategies for a better chance of success.

## Background

Obesity is a health problem that has become a worldwide epidemic over the past few decades. Several co-morbidities are commonly related with obesity such as diabetes, hypertension, osteoarthritis and cardiovascular diseases [1]. Excessive fat deposits particularly in the abdominal region impose a higher risk because this pattern of fat deposition is a stronger predictor of cardiovascular diseases and type 2 diabetes mellitus than general obesity [2, 3]. Co-morbidities are defined as the simultaneous presence of two chronic diseases or conditions in a patient [4]. Co-morbidities are associated with worse health outcomes, more complex clinical management, and increased health care costs [5]. Compared to body weight and Body Mass Index (BMI), body fat is most important in determining an individual’s risk of developing co-morbidities [6]. Study shows some of the body weight loss most probably was muscle and it effect in reducing metabolic rate [7]. Other studies show that fat loss conferred more health benefits, including a reduced risk of cardiovascular disease, type 2 diabetes and other chronic diseases, as well as maintaining long term health [2, 3, 8].

A multidisciplinary approach has identified a combination of balanced dietary intake, regular exercise and psychological intervention as potentially effective weight loss strategies in overweight and obese adults [9]. Weight loss between 5 to 10% due to lifestyle modification helps in reducing the risk of cardiovascular disease [10]. Although body weight and Body Mass Index are frequently used methods to measure the level of obesity, body compositions are also always being measured in weight loss interventions. Past studies have shown that a decrease in body weight and body fat can give potential health benefits to individuals [11–15].

There are several methods of measuring body composition including bioelectrical impedance (BIA), dual-energy X-ray absorptiometry (DEXA), and skin fold thickness [16]. Comparing between the methods of measuring body composition, BIA is the most cost-effective method of measuring body composition. As it is portable and easier to use than other technologies [17], BIA was therefore used in this study to measure body composition.

Most weight management intervention studies have reported a successful improvement of intervention on body weight at a range of 2–4 kg [18, 19] and fat mass 1.3–3.0 kg [19–21]. Although there have been many intervention studies done in multiple populations, only a few were carried out on overweight and obese women with comorbidities, especially in Malaysia. The National Health and Morbidity Survey 2011 shows prevalence of obesity among women was higher at 29.6% compared to 25.0% in males, while by category of occupation; housewives had the highest levels of obesity at 20.3%, compared to other types of occupation [22].

This paper aimed to determine changes in body composition between obese housewives without co-morbidities and with co-morbidities after they participated in a weight loss intervention. Findings from this study will help to improve strategies in weight management among those with co-morbidities.

## Methods

This is a follow-up study of the MyBFF@home, [23] which involved residents living in the People’s Housing Project (PHP) and low cost flat (LCF) around the Klang Valley. A total of 126 housewives living in 5 PHP / LCF clans in the Klang Valley were recruited for this study. This study was using a quasi-experimental design which involves pre and post intervention among the housewives. Housewives were then divided into two groups, from control group (delayed intervention from MyBFF@home Phase II) as Group 1 (G1): obesity without co-morbidities (n:84) and new recruitment for Group 2(G2): obesity with co-morbidities (n:42). Baseline socio-demographic data was obtained from month 12 of MyBFF@home phase II for Group 1: obesity without co-morbidities. A new recruitment of obesity housewives with co-morbidities was done concurrently. Co-morbidities were defined as the presence of two or more diseases or conditions in an individual [4].

The inclusion criteria was age between 18 to 59 years old, overweight and obese with the Body Mass Index (BMI) ≥ 25.0 – ≤ 39.9kgm-2, has been a housewife for at least 6 months, and can communicate in Malay or English language. Pregnant or breastfeeding mothers, as well as those with a limited ability to perform physical activity were excluded from this study. In addition, those with uncontrolled chronic illnesses (diabetes, hypertension and hypercholesterolemia) and were under a weight-loss programs or consuming products aimed at losing weight at the time of the study, were also excluded. Uncontrolled diabetes, hypertension, and hypercholesterolemia refers to fasting blood sugar levels that were constantly higher than FBS > 7.2 mmol/L used in the study, since we are being specific with blood pressure and cholesterol values despite being on medication, blood pressure > 140/90 mmHg, and cholesterol > 5.7 mmol/dl respectively [24].

**Fig. 1:**
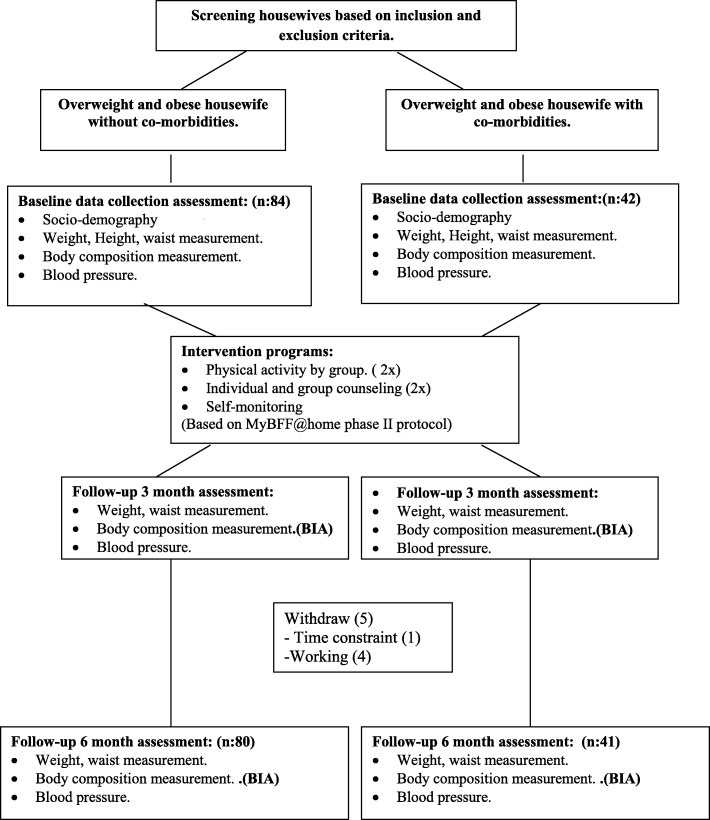
Data collection of Body composition

Information on physical activity/exercise and dietary intake was assessed using self-report questionnaires. Trained research officers with Dietetic or Nutrition backgrounds explained the questionnaire to participants and took anthropometric measurements. Both groups received a 6-month intervention (July–December 2015) consisting of diet control, physical activity and self-monitoring that used the same method in MyBFF@home phase II. The individual exercise routine for each participant consisted of a brisk walk for 30 to 45 min every day, pillow dumb bell exercise for 30 min a day, and group exercise 2 times a month. Low calorie and low fat diets were implemented in this study and self-monitoring was done 3 days a week via a food diary and a physical activity diary [23] (Fig. 1).

Body composition was measured 3 times throughout the study at baseline, 3 months, and 6 months. Body composition was measured using Bio-electrical Impedance Analyzer (BIA) (In-Body 720.) Housewives were reminded to fast the night before they were due for body composition measurement and not to wear any jewelry, watches or other objects that can affect the BIA machine reading. Bioelectrical impedance analysis (BIA) is a commonly used method for estimating body composition, and in particular body fat. BIA actually determines the electrical impedance, or opposition to the flow of an electric current through body tissues which can then be used to estimate total body water (TBW), which can be used to estimate fat-free body mass and, by difference with body weight, body fat. BIA is considered reasonably accurate for measuring groups, or for tracking body composition in an individual over a period of time, but is not considered sufficiently precise for recording of single measurements of individuals [14–16, 25].

### Statistical analysis

Descriptive analysis was done for socio-demography data. Paired T-test was used to see the changes between pre and post results of body composition while Independent T–test was used to see the mean difference between these two groups. Baseline, 3 month and 6 month data from the body composition using Bioelectrical Impedance analysis were compared using repeated measures ANOVA. Differences were considered significant at *p* < 0.05. Analysis was conducted using SPSS software (version 21.0).

## Results

Table 1 shows the socio-demographic characteristics of both groups. A total of 126 housewives were recruited a baseline but only 121 housewives completed the intervention study. Busy schedules and new jobs were reasons given by the 5 housewives who withdrew from this intervention study. Mean age for G1 was 44.3 and G2 was 51.0 respectively. The majority of G1 participants were aged between 40 and 49 years old (48.8%) and 50–59 years old for G2 (64.3%). Malay ethnicity showed a higher percentage (92.9%) compared with other ethnic groups. In addition, most of the participants G1 were in the overweight category (25–29.9 kg/m^2^), whereas most of the G2 participants were in the obesity stage I (30–34.9 kg/m^2^) category.

**Table 1:** Socio demographic characteristic of the obesity with and without Co-morbid housewives group. (N:126)

Socio-demography characteristic	Obesity (G1) n:84		obesity with co-morbidities (G2) n:42		*p*-value
	n	%	n	%	
Age					
• 18–29	7	8.3	1	2.4	
• 30–39	13	15.5	1	2.4	0.001*
• 40–49	41	48.8	13	31	
• 50–59	23	27.4	27	64.3	
Ethnicity					
• Malay	78	92.9	39	92.9	
• Chinese	–	–	1	2.4	0.800
• Indian	6	7.1	2	4.8	
Marital status					
• Single	2	2.4	–	–	0.196
• Married	78	92.8	35	83.3	
• Widow	4	4.8	7	16.6	
Education level					
• Primary	11	13.1	12	28.6	
• Secondary	60	71.5	12	28.6	0.168
• Tertiary	9	10.7	14	33.3	
• Others	4	4.8	4	9.5	
Body Mass Index (BMI)					
• 25–29.9	45	53.6	17	40.5	
• 30–34.9	22	26.2	20	47.6	0.660
• 35–39.9	17	20.2	5	11.9	
Non-communicable diseases					–
• Diabetes Mellitus			11	26.2	
• Hypertension			14	33.3	
• Hyperlipidemia			5	11.9	
• Combined disease			12	28.6	

A two-way repeated measure ANOVA (RM-ANOVA) was conducted to compare the effect of (IV) time of intervention from baseline to six months on (DV) body composition changes before, during and after the intervention. There was a significant effect of time of intervention on body composition changes, F (2,238) =3.778, *p* = < 0.05. Pairwise comparison with confidence interval adjustment was performed. The results showed that there were significant differences in one comparison of time in body fat: Baseline to month 6 (mean difference: 0.88,95% CI: 0.10,1.66) for G1 and Baseline to month 3 (mean difference:1.15, 95% CI:0.36,1.94) for G2. Similar results were found in fat mass between group G1 and G2 in which there were significant differences in baseline to month 3 of time. Both group showed significant differences, (mean difference: 0.64, 95% CI: 0.05,1.24) for G1 and (mean difference: 0.71, 95% CI: -0.01,1.42) for G2. Only G1 showed a significant difference in two comparison of time for visceral fat area, baseline to month 3 (mean difference: 11.28, 95% CI: 2.40, 20.15) and month 3 to month 6 (mean difference: -9.22, 95% CI: -17.87,-0.56). As indicated from RM-ANOVA, there were no significant differences between group for body fat, fat mass and skeletal muscle regardless of time (*p* > 0.05). However, there was a significant difference in mean visceral fat between these 2 groups with mean difference of 11.49(95% CI: -20.07, − 2.91) (*p* < 0.05) (Table 2).

**Table 2:** Comparison of body composition measurement within and between obesity without co-morbid group and obesity with co-morbid housewives group based on time and group (Time and group effect)

Parameter	Comparison	Obesity without co-morbidities (G1) n:80		Obesity with co-morbidities (G2) n:41		Comparison between (Obesity – Obesity with co-morbid)	
		Mean Difference (95%CI)	*p*-value	Mean difference (95%CI)	*p*-value	Mean difference (95%CI)	*p*-value
Body fat (%)	Baseline – Month 3	0.56(−0.03, 1.16)	0.070	1.15(0.36,1.94)	0.002*	− 1.02 (− 2.83, 0.80)	0.270
	Month 3 – Month 6	0.32(−0.47,1.10)	0.980	−0.24(− 1.47,0.98)	1.000		
	Baseline – Month 6	0.88(0.10,1.66)	0.020*	0.91(− 0.11,1.93)	0.096		
Fat mass (kg)	Baseline – Month 3	0.64 (0.05,1.24)	0.030*	0.71(− 0.01,1.42)	0.051	−0.90 (− 3.60, 1.82)	0.516
	Month 3 – Month 6	− 0.14 (− 1.07,0.78)	1.000	− 0.19(− 1.19,0.80)	1.000		
	Baseline – Month 6	0.50 (− 0.32,1.32)	0.414	0.52(− 0.28,1.31)	0.340		
Skeletal muscle mass (kg)	Baseline – Month 3	0.04(−0.35,0.44)	1.000	−.06(−.52,0.41)	1.000	0.52(−0.548,1.580)	0.339
	Month 3 – Month 6	−0.16 (− 0.56,0.24)	0.990	0.05(− 0.55,0.65)	1.000		
	Baseline – Month 6	−0.12 (−.0.38,0.14)	0.830	−0.01(− 0.38,0.37)	1.000		
Visceral fat area (cm^2^)	Baseline – Month 3	11.28(2.40,20.15)	0.008*	−9.32 (− 24.64,5.99)	0.409	−11.49(− 20.07, − 2.91)	0.009*
	Month 3 – Month 6	−9.22(−17.87,-0.56)	0.033*	9.69(−5.46,24.84)	0.354		
	Baseline – Month 6	2.06(−0.84,4.96)	0.258	0.37(− 2.73,3.46)	1.000		

-Pairwise comparison with confidence interval adjustment, *significant at *p* < 0.05

-Adjustment for multiple comparison: Bonferroni

There was a significant difference of mean visceral fat area between G1 and G2 based on time (F = 8.560, *p* = 0.003). It showed that the intervention programs had the most effect on visceral fat area compared to other components of body composition. In addition, the obesity without co-morbidities group showed a larger decrease in mean visceral fat area compared to the obesity with co-morbidities group (Table 3).

**Table 3:** Comparison of body composition measurement within-between obesity with and without co-morbid housewives based on time (Intervention effect)

Parameter	Visit	Group	Mean	Intervention effect (Time* Group) 95% CI		F	*P*-value
				lower	upper		
Body fat (%)	Baseline	Obesity	44.3	43.23	45.27	0.843	4.201
		Obesity with co-morbidities	45.5	44.04	46.90		
	Month 6	Obesity	43.4	42.22	44.52		
		Obesity with co-morbidities	44.6	42.95	46.17		
Fat mass (kg)	Baseline	Obesity	31.7	30.15	33.30	0.009	0.981
		Obesity with co-morbidities	32.6	30.44	34.85		
	Month 6	Obesity	31.2	29.62	32.82		
		Obesity with co-morbidities	32.1	28.89	34.36		
Visceral fat area (cm^2^)	Baseline	Obesity	115.8	111.20	120.44	8.560	0.003*
		Obesity with co-morbidities	119.9	113.41	126.34		
	Month 6	Obesity	113.76	108.92	118.59		
		Obesity with co-morbidities	119.5	112.75	126.27		
Skeletal muscle mass (kg)	Baseline	Obesity	21.2	20.55	21.84	0.359	0.656
		Obesity with co-morbidities	20.7	19.78	21.58		
	Month 6	Obesity	21.3	20.68	21.94		
		Obesity with co-morbidities	20.7	19.80	21.57		

*significant at *p < 0.05*

## Discussion

In our study, we found that a six-month weight reduction intervention produced a significant reduction and improvement in body composition among obese without co-morbidities and obese with co-morbidities housewives. A combination of physical activity, dietary modification and also self-monitoring was used in our study to see the effect and changes toward body composition, consist of body fat, fat mass, visceral fat and skeletal muscle mass between obesity without co-morbidities and obesity with co-morbidities group. Our dietary intervention was adapted from MyBFF@home phase II, with the addition of dietary management for Type 2 diabetes mellitus, hypertension and hypercholesterolemia, whereas exercise and physical activity was based on common activities that were convenient and can be easily done by housewives [23]. Many studies used this combination in order to achieve positive and good results. Although the type of exercise and diet control were different in certain studies but the concepts of intervention were the same to see which approach was more suitable for certain subjects based on ages, gender, and cultures [26, 27].

There was a difference in age range of both groups where G2 participants were generally older than those in G1. This will confound results because as age increases, metabolic processes decrease. Regulation of energy metabolism occurs during normal aging and this also affects progression of body weight and body composition reduction [28]. In regards to BMI status, G1 participants were predominantly overweight (BMI range 25.0–29.9 kg/m2) while G2 participants were mainly obese (BMI range 30.0–34.9 kg/m2). This also might affect the result of body composition between both groups in addition to other factors such as age. Although we can predict the outcome, G2 also have shown positive results in reduction of the parameter measured.

Our findings demonstrated reductions in mean percentages of body fat, fat mass, and visceral fat area in both groups. In contrast, skeletal muscle mass increased in G1 participants but was stagnant in G2 participants over the 6-month intervention period. Among G1 participants, decreasing patterns were found in body fat percentage with mean difference of 0.88%, fat mass of 0.50 kg and visceral fat area of 2.06 cm2. Similar to our study, other studies also reported a decreasing pattern in body fat percentage with mean difference of 0.4% [14] and fat mass with mean difference of 1.3 kg [20] within 4 months of undergoing dietary and exercise intervention. Other weight reduction intervention studies conducted over 12 months also reported significant decreases in body fat percentage [15, 29, 30]. However some of the previous studies did not include self-monitoring or behavioral modification, which are two of the most important elements in weight loss intervention studies.

The most important finding between both groups was changes in visceral fat area which showed a significant mean difference of − 11.49 cm2 (p- value = 0.009). This difference is most probably due to differences in body mass index between both groups as mentioned before. As discussed earlier G2 participants were obese compared to G1 participants who were mostly overweight. The degree of fat accumulation between both groups also differs, where G2 would have more accumulated more fat than G1. Other studies report that type 2 diabetes is obesity-dependent and that obesity is the main etiological cause of type 2 diabetes [31, 32]. Accumulation of intra-abdominal or visceral fat is associated with insulin resistance and is a major feature of metabolic syndrome, which increases one’s risk of developing diabetes and cardiovascular disease (CVD) [33]. Comparing the overall results of this study between these two groups, it seems that a slower progression is seen in G2 compared to G1. Other studies reported similar findings where obese subjects with co-morbidities such as Type 2 diabetes mellitus had difficulties in reducing body weight and other parameters as compared to obese subjects’ co-morbidities [12].

Lastly, the results in this study was also possibly affected by adherence and commitment of housewives to the prescribed physical activity routines and recommended diets. From this study not all housewives attended every follow-up but most managed to complete the 6-month intervention period. Only 5 housewives withdrew in the middle of the study. It is important to regularly follow-up on every instruction given to them and this study also aimed to empower housewives to reduce body weight in order to gain successful results.

The strength of this study was the use of a combined approach in the intervention package including dietary modification, physical activity and self-monitoring. In addition, findings from this study will contribute to the literature on the impact of obesity with co-morbidities among housewives in Malaysia. Although this study only focused on a singular urban area in a central part of Peninsular Malaysia, it can nonetheless be extended to rural areas in other parts of Peninsular and East Malaysia for future studies.

## Conclusions

Following a 6-month intervention period, obese participants without co-morbidities showed more significant improvements in body fat percentage, fat free mass and visceral fat area compared to obese participants with co-morbidities. In addition, a combination of healthy eating interventions, regular physical activity and lifestyle modification is needed to more efficiently manage weight loss and improve metabolic parameters.

## Abbreviations

ANOVA: Analysis of Varian
BIA: Bio-electrical Impedance Analyzer
CVD: Cardiovascular Disease
DEXA: Dual-energy X-ray absorptiometry
LCF: Low cost flat
MyBFF@home: My Body is Fit and Fabulous at home
PHP: People’s Housing Project
RM-ANOVA: Repeated Measure ANOVA
SPSS: Statistical package for social science
TBW: Total body water
